# Structure and molecular function of the BCL7 proteins

**DOI:** 10.1042/BST20250117

**Published:** 2026-06-17

**Authors:** Franck Martin, Elisa Bergamin

**Affiliations:** 1 Department of Functional Genomics and Cancer & Department of Integrated Structural Biology, Institut de Génétique et de Biologie Moléculaire et Cellulaire (IGBMC), Illkirch Cedex, France; 2 Université de Strasbourg, Strasbourg, France; 3 Centre National de la Recherche Scientifique UMR 7104, Illkirch Cedex, France; 4 Institut National de la Santé et de la Recherche Médicale U1258, Illkirch Cedex, France

**Keywords:** BCL7, cancer, chromatin, cryo-EM, nucleosome, SWI/SNF

## Abstract

Initially identified through chromosomal translocations in lymphomas, the BCL7 protein family, comprising the three paralogues BCL7A, BCL7B, and BCL7C, has recently emerged as a core component of mammalian SWI/SNF ATP-dependent chromatin remodeling complexes. Although their functions remained poorly understood for many years, recent structural and biochemical studies have substantially improved our understanding of their roles. Cryo-electron microscopy studies revealed that BCL7 proteins interact with nucleosomes through a conserved N-terminal arginine anchor motif that binds the nucleosomal acidic patch and stabilize the actin-related protein module through a conserved β-hairpin motif. These findings identify BCL7 proteins as structural elements that contribute to nucleosome engagement and SWI/SNF complex integrity. Comparative analyses further suggest that key structural features of BCL7 proteins are conserved across evolution despite limited sequence similarity. In addition to their roles in chromatin remodeling, increasing evidence links BCL7 proteins to hematological malignancies, solid tumors, and developmental disorders, highlighting their emerging value as biomarkers and potential therapeutic targets. This review summarizes current knowledge on the structure, evolution, and functions of the BCL7 family and outlines future directions for elucidating their contribution to chromatin regulation and disease.

## Discovery of BCL7 proteins

### Genomic organization

**Figure 1:**
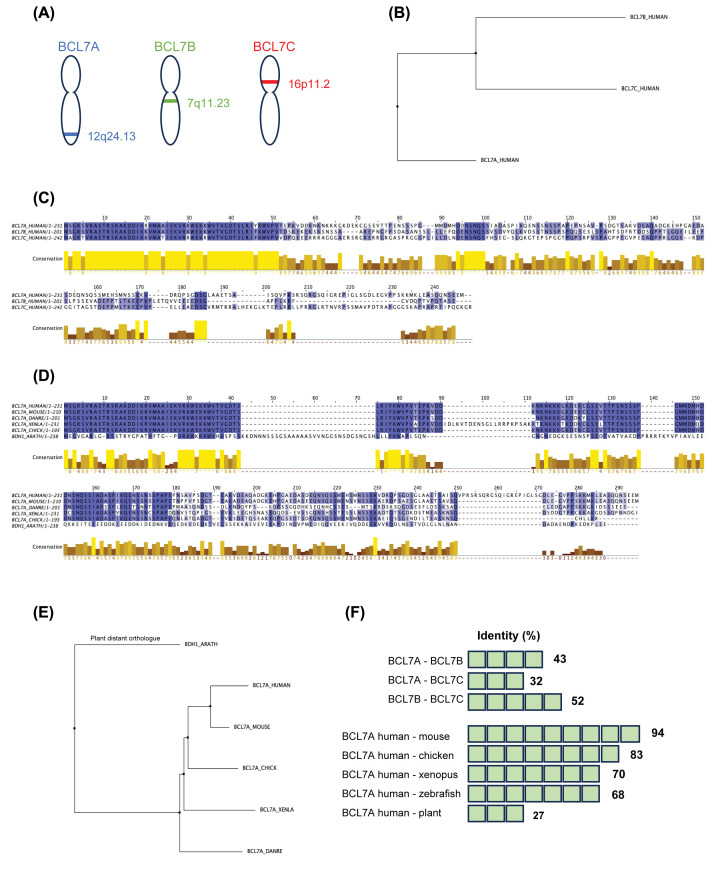
Evolutionary conservation and organization of the BCL7 protein family (**A**) Genomic organization and chromosomal localization of the three human BCL7 paralogues, BCL7A, BCL7B, and BCL7C. (**B**) Phylogenetic relationship between the three human BCL7 family members. (**C**) Multiple sequence alignment of human BCL7A, BCL7B, and BCL7C proteins highlighting the strong conservation of the N-terminal region and the greater divergence of the remaining protein sequence. Conservation scores are shown below the alignment. (**D**) Multiple sequence alignment of representative BCL7A orthologues from vertebrate species together with the plant protein BDH1, illustrating conservation of the N-terminal region despite reduced overall sequence similarity. Conservation scores are shown below the alignment. (**E**) Phylogenetic tree of representative BCL7 orthologues generated using the neighbor-joining analysis based on BLOSUM62 substitution scores. BDH1 is included as a distant plant orthologue. (**F**) Pairwise sequence identity comparison between BCL7 family members and representative BCL7 orthologues relative to human BCL7A.

The discovery of the BCL7 protein family began with the identification of B-cell lymphoma 7 protein family member A (BCL7A), a gene disrupted in chromosomal translocations associated with high-grade B-cell lymphoma, an aggressive type of non-Hodgkin lymphomas. Chromosomal translocations are a hallmark of malignancy and in lymphomas they frequently involve the immunoglobulin heavy chain (*IGH*) locus at chromosome 14q32.33, resulting in deregulated expression of oncogenes [1,2]. A new three-way translocation was discovered where IGH and MYC had become juxtaposed with an unknown sequence on chromosome 12q24.1 (Figure 1A), which led to the identification of a previously uncharacterized gene: BCL7A [2,3].

Initial sequence analysis of BCL7A revealed no canonical domains. However, it was reported that BCL7A exhibits some homology to caldesmon, an actin-binding protein [3]. Subsequent genomic sequencing efforts identified two additional BCL7-related transcripts: BCL7B, localized on chromosomes 7q11.2; and BCL7C, localized on chromosome 16p11 (Figure 1A). Both transcripts share significant homology with the N-terminal region of BCL7A [2]. All three BCL7 family members are ubiquitously expressed in human tissues, although their precise biological roles remained elusive for many years. Recently, emerging links to chromatin regulation and cancer and their evolutionary conservation have renewed scientific interest [4].

### BCL7 proteins as integral components of the SWI/SNF complex

Biochemical and large-scale proteomic studies confirmed that BCL7 proteins are integral components of the mammalian SWI (mSWI/SNF) complex [5,6], a multi-protein chromatin remodeling complex that utilizes the energy derived by ATP hydrolysis to reorganize chromatin, regulating chromatin structure and gene expression [7]. BCL7 proteins are core components of all the major types of mSWI/SNF complexes: BRG1-associated factor (cBAF), polybromo-associated BAF (PBAF), and non-canonical BAF (ncBAF) but are not present in yeast SWI/SNF complexes.

### Evolutionary conservation across species

The three BCL7 genes are paralogs, thought to have arisen from ancient gene duplication events. They are proteins of approximately 25 kDa and consist of 231 amino acids in BCL7A, 202 in BCL7B, and 242 in BCL7C. Pairwise sequence comparisons between human BCL7 paralogues reveal moderate overall sequence identity (32%–53%). While their N-terminal region (encompassing the first 51 amino acids) is highly conserved, consistent with a shared functional core across the family, the C-terminal region is substantially more divergent, suggesting paralogue-specific adaptations or regulatory specializations (Figure 1B,C,F).

Phylogenetic analyses reveal that BCL7 genes are unique to multicellular eukaryotes, with orthologues of BCL7A, BCL7B, and BCL7C identified in mammals, birds, amphibians, and fish. Consistent with their evolutionary conservation, BCL7A displays high sequence identity across vertebrates, including ∼94% between human and mouse, ∼83% with chicken, ∼70% with Xenopus, and ∼68% with zebrafish (Figure 1D–F). This distribution supports the idea that the BCL7 family emerged to accommodate the increased regulatory complexity associated with multicellularity.

Beyond vertebrates, structurally related proteins have been identified in other eukaryotic lineages. BCL-domain homologs 1 and 2 (BDH1/2), shared subunits of plant SWI/SNF complexes, have been proposed as distant orthologs of mammalian BCL7 proteins (∼27% identity with plant BDH1 (Figure 1D–F)) based on large-scale phylogenomic and proteomic analyses [8,9].

Together, these observations highlight that while vertebrates evolved three specialized BCL7 paralogues, plants retained structurally related factors that likely fulfill conserved architectural and regulatory roles in SWI/SNF stability and chromatin accessibility.

## Structural organization of the BCL7 proteins

The elucidation of how the BCL7 proteins operate within SWI/SNF chromatin-remodeling complexes has been substantially advanced by recent breakthroughs in structural biology.

**Figure 2:**
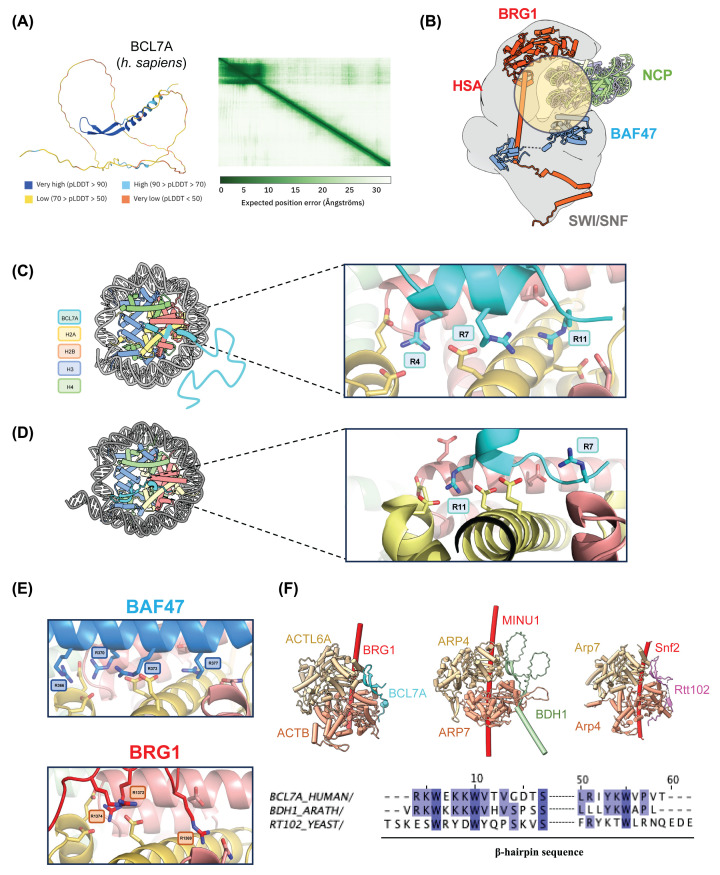
Structural organization and molecular interactions of BCL7 proteins within SWI/SNF complexes (**A**) AlphaFold prediction of human BCL7A and corresponding predicted aligned error matrix showing a largely disordered protein with a structured N-terminal region. (**B**) Schematic localization of BCL7 within the mSWI/SNF complex at the interface between the nucleosome (NCP), ATPase module, and actin-related protein (ARP) module. (**C**) Structure of BCL7A bound to the nucleosome showing interaction of the N-terminal region with the acidic patch through conserved arginine residues (R4, R7, and R11). (**D**) Structure of BCL7A within the ncBAF complex illustrating an alternative acidic patch interaction mode and conformational flexibility of the N-terminal region. (**E**) Acidic patch interactions mediated by BAF47 and BRG1, highlighting conserved arginine-based nucleosome recognition mechanisms within SWI/SNF complexes. (**F**) Structural comparison of β-hairpin-mediated ARP interactions involving BDH1, Rtt102, and BCL7A. Sequence alignment highlights conservation of key residues despite limited sequence similarity.

Sequence-based analyses, using DISOPRED3 [10] and AlphaFold [11] circular dichroism and NMR experiments [12], all predict that the majority of the protein’s length is disordered. Only the conserved N-terminal region is predicted to have some structure. AlphaFold modeling [13–15] indicate that this conserved segment consists of an α-helix spanning the first 30 residues, followed by two antiparallel β-strands within the next 20 residues (Figure 2A). Electrophoretic mobility shift assays and biolayer interferometry experiments [12] have demonstrated that all three BCL7 paralogs bind nucleosomes with similar affinity (∼200 nM), and that the interaction is mediated by their conserved N-terminal region [12,16].

Current topological information on BCL7 proteins primarily derives from cross-linking mass spectrometry studies, which identified interactions with the BRG1 (SMARCA4) ATPase and the BAF47 (SMARCB1) subunit, two core components that directly engage the nucleosome and are essential for SWI/SNF remodeling activity. Additional interactions with histone H2B, DPF2, and actin have also been reported [5–7,12,17], placing BCL7 proteins at the interface between the nucleosome, the ATPase module, and the ARP module (Figure 2B).

Despite major advances in the cryo-electron microscopy (cryo-EM) characterization of BAF and PBAF complexes [18–21], the precise localization of BCL7 has long remained unresolved. In several structures, BCL7 was either absent from reconstituted assemblies or poorly resolved, likely reflecting intrinsic conformational flexibility or sub-stoichiometric incorporation within the complexes [20,21]. More recently, integrated structural and biochemical studies have substantially refined our understanding of BCL7 function, revealing its direct contribution to nucleosome engagement and clarifying its architectural role within SWI/SNF assemblies [12,22,23].

### Anchoring the remodeler: BCL7 proteins at the nucleosome interface

The high-resolution cryo-EM structure of the BCL7A–nucleosome complex [12] revealed that the first 30 amino acids of BCL7A form the main interface responsible for nucleosome binding. This conserved N-terminal region adopts two short α-helices (α1 and α2) connected by a flexible linker (Figure 2C). Within the first helix, three arginine residues (R4, R7, and R11) play a central role in recognizing the nucleosomal acidic patch, a negatively charged surface formed by histones H2A and H2B that serves as a key docking platform for many chromatin-associated factors. Each arginine inserts into a slightly different pocket within this acidic region, establishing a network of electrostatic and hydrogen-bond interactions that collectively stabilize BCL7A binding to the nucleosome. According to the classification proposed by McGinty and Tan [24,25], R4 classifies as canonical arginine anchor, R7 is a type 2 arginine anchor binding the cavity right next to the canonical arginine binding cleft and R11 is a type 1 variant arginine. Together, these interactions create a multivalent binding interface that likely strengthens nucleosome association and contributes to stable recruitment of SWI/SNF complexes to chromatin.

The α2 helix of BCL7A lies across the H2B C-terminal α-helix and the H2A α1 helix, extending toward the phosphate backbone of nucleosomal DNA near superhelical location +4.5 (SHL +4.5). Although modeled as a poly-alanine chain due to local resolution limitations, this orientation points to a potential secondary contact site involving DNA and histone components, including possible contributions from the H2B and H2A histone tail, which may participate in stabilizing BCL7A at the nucleosome interface [12].

Interestingly, similar densities have been observed at this location in other chromatin-associated complexes. In the NuA4 complex (via Epl1) and the RSC complex (via Sth1), additional density is also detected across the H2B C-terminal α-helix and H2A α1 helix [26,27]. These observations suggest that secondary contact sites point toward a recurring feature of chromatin-interacting complexes. However, the molecular nature of this interface remains unclear and warrants further investigation, particularly regarding the potential contribution of the H2A histone tail and the role of post-translational modifications.

Additional structures of BCL7A and BCL7B have recently been resolved in the context of the ncBAF complex, revealing distinct conformations of the BCL7 N-terminal helix at the nucleosome H2A–H2B acidic region [22,23]. In the BCL7A–NCP–ncBAF structure (Figure 2D), R11 occupies the canonical acidic patch pocket, while R7 engages an adjacent variant site, suggesting a reduced contribution of R4 in this context [22]. In contrast, the BCL7B–ncBAF structure [23] displays a conformation more similar to that observed for isolated BCL7A bound to the nucleosome [12], with R4 positioned in the canonical pocket and R7 and R11 contacting neighboring variant sites. Notably, the N-terminal helix appears longer (residues ∼2–18) in this structure [23].

Together, these observations indicate that the N-terminal helix of BCL7 proteins exhibits conformational flexibility, potentially adopting distinct binding modes depending on the molecular context [12,22,23]. This plasticity may reflect a dynamic equilibrium between disordered and helical states, allowing BCL7 to adapt its nucleosome interaction within different SWI/SNF assemblies.

### Arginine anchors as a unifying principle of SWI/SNF–nucleosome binding

Cryo-EM and biochemical studies of the SWI/SNF complex have shown that BRG1 engages the nucleosome on its upper face [19], whereas BAF47 [18–20] binds on the opposite side (Figure 2E). Within BRG1, a loop in the Snf2 ATP-coupling (SnAc) domain contains three arginine residues (R1369, R1372, R1374) that interact with the nucleosomal acidic patch (Figure 2C). In contrast, BAF47 contains an α-helix harboring four arginine residues (R366, R370, R373, R377), which also target the same acidic region. BRG1 establishes extensive interactions with the nucleosome through its ATPase motor lobes, which bind nucleosomal DNA [20]. Despite differences in the number and spatial arrangement of arginine residues, the overall mode of interaction with the acidic interface appears to be highly conserved (Figure 2C).

It is interesting to note that the presence of arginine-binding motifs appears to be a recurring feature among SNF2-type chromatin remodelers such as ISWI or RSC, but also for several chromatin-associated complexes such as SAGA and NuA4 acetyl transferases, reflecting the central role of the acidic region of the nucleosome as a preferred interaction site [26,28,29].

### The β-hairpin fold is an evolutionarily conserved Actin/ARP interacting module in SWI/SNF complexes

Structures of BCL7A and BCL7B within the ncBAF complex [22,23] show that residues 26–51 form an antiparallel β-sheet that stabilizes the ARP module by bridging ACTL6A and β-actin (Figure 2F). This interface is maintained through an extensive network of hydrophobic interactions involving conserved aromatic residues of BCL7A/B, together with hydrogen bonds and salt bridges formed with both ACTL6A and β-actin. Conformational differences observed in the ADP-bound state support the idea that this region retains a degree of structural flexibility, potentially linked to nucleotide-dependent rearrangements within the ARP module. Together, these findings indicate that the β-hairpin motif is critical for BCL7 incorporation and contributes to the structural integrity of the SWI/SNF complex. Consistently, a recent study identified the β-hairpin-containing region as essential for stable integration of BCL7 into SWI/SNF assemblies [16]. However, considering the current cryo-EM structures of the BAF and PBAF complexes (references), it is hard to envision how BCL7 would be able reach the ARP module in this context given that the ARP module is about 40 Å away from the nucleosome.

Interestingly, despite the absence of sequence conservation, structurally related β-hairpin motifs appear to be preserved across evolution. Plant BDH proteins contain a short conserved region predicted to adopt a similar fold (Figure 2F), suggesting conservation of key structural determinants required for ARP module association [8]. Likewise, the yeast SWI/SNF subunit Rtt102 displays a structurally analogous β-sheet architecture and binds the ARP module through a comparable interface [30]. Together, these observations support the idea that chromatin remodeling complexes preserve core ARP-stabilizing architectures through structural rather than sequence conservation.

### A flexible regulatory platform: the unexplored C-terminal regions of BCL7 proteins

In contrast to the highly conserved N-terminal region involved in nucleosome and ARP-module interactions, the C-terminal regions of BCL7 proteins remain poorly characterized and are predicted to be intrinsically disordered. Such disordered regions are common among chromatin-associated proteins and often function as flexible platforms for transient and context-dependent interactions [31]. The strong sequence divergence observed between the C-terminal regions of BCL7A, BCL7B, and BCL7C suggests that these domains confer paralogue-specific regulatory functions while preserving a conserved structural core.

These flexible regions may mediate dynamic interactions with histones, DNA, SWI/SNF subunits, or lineage-specific regulatory factors, thereby contributing to the functional plasticity of SWI/SNF complexes. In addition, they are likely to represent sites for post-translational modifications, potentially modulating BCL7 stability, chromatin affinity, or complex assembly in response to cellular signaling [32]. Their amino acid composition also raises the possibility that BCL7 proteins contribute to liquid–liquid phase separation [12,33] mechanisms involved in chromatin organization, similarly to other intrinsically disordered chromatin regulators such as HP1 and EZH2 [34,35]. Although experimental evidence remains limited, the disordered C-terminal regions of BCL7 proteins may therefore provide an important regulatory interface linking chromatin remodeling to nuclear organization and cell-type-specific gene regulation.

## Functional roles of BCL7 proteins in chromatin remodeling and cancer

### Functional contributions of BCL7 proteins to chromatin remodeling

BCL7 proteins are integral components of the mSWI/SNF chromatin-remodeling complex, where they support efficient nucleosome remodeling and are required for full enzymatic activity [12]. Together with BRG1, they contribute to chromatin remodeling and large-scale changes in gene expression [35]. Recent studies suggest that BCL7A also influences mitochondrial function, albeit indirectly through its regulatory role in chromatin dynamics. For instance, decreased BCL7A expression has been associated with modifications in mitochondrial metabolism and reactive oxygen species production [36].

Recent studies indicate that the loss of BCL7A does not compromise the overall integrity of the SWI/SNF complex, but rather affects its genomic engagement [12,16]. BCL7A contributes to mSWI/SNF remodeling activity by promoting BRG1 occupancy at selected promoters and enhancer regions, suggesting a role in stabilizing SWI/SNF interactions with nucleosomal H2A–H2B dimers [12,17,37]. Other studies show that the N-terminal α-helix of BCL7A (arginine anchor) is important in modulating cBAF chromatin remodeling and for its tumor-suppressive activity in diffuse large B-cell lymphoma (DLBCL) [16].

Functionally, BCL7B depletion leads to dysregulated signaling characterized by increased Notch pathway activation and suppression of Wnt signaling, which collectively bias neural progenitor cell differentiation toward gliogenic lineages. These findings highlight the important role of BCL7B in regulating pathways such as Wnt signaling, underscoring its broader relevance in both normal cellular processes and cancer progression [37–39].

Dysregulation of the BCL7 subunits of the mSWI/SNF chromatin remodeling complex has been linked to multiple cancers, reflecting their diverse and context-dependent roles in tumorigenesis. Although structurally related, BCL7A, BCL7B, and BCL7C display distinct, sometimes opposing functions across cancer types.

### BCL7A in hematological malignancies and chromatin instability

**Figure 3:**
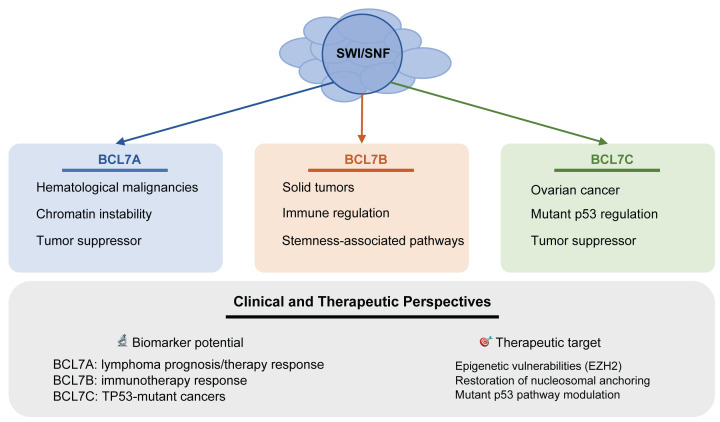
Functional and pathological roles of BCL7 proteins in cancer Schematic overview of the distinct functions of BCL7 paralogues within the SWI/SNF chromatin remodeling complex and their involvement in cancer-related processes. The figure highlights the different biological pathways and tumor-associated functions linked to BCL7A, BCL7B, and BCL7C, together with their potential clinical and therapeutic implications.

BCL7A is labeled as a ‘cancer gene’ by the COSMIC database as it is frequently mutated in a variety of cancers and is an established tumor suppressor (Figure 3) [40,41]. For instance, BCL7A is frequently mutated in DLBCL [42,43]. Loss of BCL7A expression has been associated with poor prognosis in these malignancies, suggesting its critical role in maintaining normal cellular functions and preventing oncogenic transformation [44]. Conversely, in Hodgkin’s lymphoma, BCL7A has been identified as a gene of interest due to its potential involvement in Reed-Sternberg cell pathogenesis [45]. For example, hypermethylation of the BCL7A promoter has been shown to lead to its inhibition in acute myeloid leukemia, which can be reversed using DNA methyltransferase inhibitors [46].

### BCL7B in solid tumors and immunity

Recent studies have highlighted the dual role of BCL7B in cancer (Figure 3). For example, it has been associated with poor prognosis in several cancers, including glioblastoma multiforme, glioma, and oral squamous cell carcinoma, where high expression levels correlate with poorer survival outcomes [47]. BCL7B is involved in the regulation of apoptosis, suggesting that it may contribute to maintaining cellular homeostasis by preventing uncontrolled cell proliferation [39,48]. On the other hand, BCL7B was reported to play an oncogenic part in pancreatic cancer [49,50]. BCL7B was also found to be involved in immune-related pathways, indicating a potential role in shaping the tumor microenvironment and anti-tumor immunity [47,51].

### BCL7C as a tumor suppressor

BCL7C has recently emerged as a potential tumor suppressor (Figure 3). Transcriptomic and functional analyses indicate that BCL7C is under-expressed in ovarian carcinomas, and this reduced expression correlates with poor clinical prognosis [52]. Mechanistically, BCL7C inhibits the activity of the mutant p53 protein, thereby limiting tumor growth and highlighting its role in preserving genomic integrity [52]. Beyond ovarian cancer, mutations and deletions in BCL7C have been documented in pan-cancer genomic databases such as TCGA and COSMIC, suggesting broader relevance across multiple cancer types [52].

### BCL7 proteins as emerging therapeutic targets

Structural studies of BCL7 proteins have transformed our understanding of their role within SWI/SNF complexes and revealed multiple therapeutic opportunities. By defining the molecular interface between BCL7 and the nucleosome, these studies enable targeted drug development, mutation-specific therapeutic strategies, and exploitation of synthetic-lethal vulnerabilities.

The frequent coexistence of BCL7A alterations with activating EZH2 mutations in DLBCL suggests that BCL7A-deficient tumors may be particularly sensitive to epigenetic therapies targeting EZH2. These observations suggest that BCL7A could potentially serve as a biomarker for patient and therapeutic response in SWI/SNF-deficient lymphomas [37]. The involvement of BCL7B in antigen presentation, immune-related signaling pathways, and tumor immune-cell infiltration indicates that it may represent a promising biomarker for immunotherapy response. In addition, its association with cancer stem-like properties and treatment resistance raises the possibility that targeting BCL7B-related pathways could help limit immune evasion and tumor recurrence [39,51]. BCL7C has recently been identified as a negative regulator of mutant p53 activity in ovarian cancer models. Reduced BCL7C expression correlates with poor prognosis and increased chemoresistance, raising the possibility that restoring BCL7C function, or mimicking its inhibitory effects on mutant p53 signaling, could represent a therapeutic strategy in TP53-mutant cancers [52]. More broadly, structural characterization of BCL7-nucleosome interactions may provide a framework for future strategies aimed at modulating SWI/SNF–chromatin engagement [12,22,23].

Because BCL7 family members are predominantly intrinsically disordered proteins, their pharmacological targeting could pose substantial challenges. Nevertheless, development of new strategies and advances in computational protein design are beginning to erode the boundaries of what has traditionally been considered *undruggable*. Molecular glues [53], deep-learning-based frameworks such as the Logo system [54] and RF-diffusion approaches [55] enable the generation of proteins capable of engaging highly flexible protein and peptide targets with high affinity and precision. These developments are examples of how the therapeutic landscape for intrinsically disordered proteins, including BCL7, is expanding, opening new venues for therapeutic opportunities.

## Conclusion

**Figure 4:**
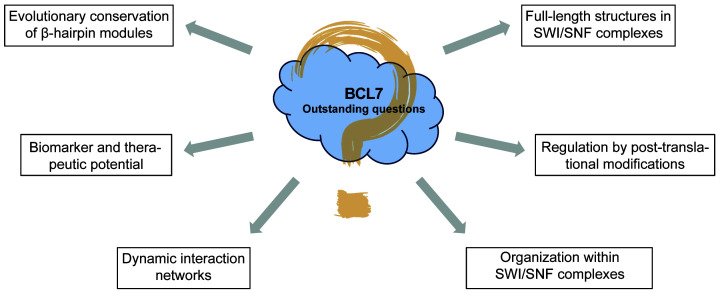
Future perspectives for BCL7 proteins Schematic representation of major open questions and unexplored aspects of BCL7 biology, highlighting unresolved mechanisms related to structural organization, regulatory pathways, interaction networks, evolutionary conservation, and potential biomarker and therapeutic applications.

Despite recent advances in the structural and functional characterization of BCL7 proteins, many aspects of their biology remain unresolved (Figure 4). A key question is how BCL7A dynamically associates within SWI/SNF complexes of the BAF and PBAF subtypes, where the presence of BAF47 may introduce competition for the acidic patch and potentially drive alternative conformational states. Investigating the topology of BCL7 proteins within BAF and PBAF complexes, and comparing these arrangements to ncBAF, will be essential to better understand their functional roles [12,35].

Despite the absence of sequence similarity, the close structural alignment between the antiparallel β-sheet of BCL7 and that of Rtt102 in the yeast ARP module highlights a striking example of structural convergence [8,22,23,30]. This conservation extends across chromatin remodeling complexes, as recent cryo-EM structures of the human SRCAP and TIP60 complexes reveal analogous assembly principles, with a similar β-sheet contributed by the DMAP1 subunit [56,57]. A comparable architecture is also observed in the yeast NuA4 complex, where Eaf2 forms a β-sheet at the interface between Act1 and Arp4 [26]. Together, these observations suggest that this β-sheet motif represents a conserved structural solution for stabilizing ARP modules, independent of sequence conservation. In this context, BCL7 likely fulfills a role analogous to these subunits, acting as a structural scaffold that reinforces inter-subunit interactions within the ARP module. More broadly, this supports the idea that chromatin remodeling complexes have evolved to preserve key architectural features through structural rather than sequence conservation, ensuring functional robustness across species and complex variants.

A promising direction concerns the post-translational regulation of BCL7 proteins, which may modulate their stability, chromatin affinity, and incorporation into different subcomplexes. No post translational modifications have been experimentally characterized in the literature to date. Modifications such as phosphorylation, ubiquitination, or acetylation of the intrinsically disordered C-terminal regions could provide a dynamic layer of control over SWI/SNF activity, linking chromatin remodeling to signaling pathways and environmental cues [12].

From a translational perspective, the frequent perturbation of BCL7 genes in cancers underscores their potential as therapeutic targets (Figure 3). Restoring or mimicking BCL7 function could help reestablish appropriate chromatin regulation in tumors characterized by SWI/SNF dysfunction. BCL7A has emerged as a new tumor suppressor gene, useful both as an independent prognostic biomarker in gliomas and as an indicator of chemotherapy response [48]. The emerging roles of BCL7B in immune regulation and cellular plasticity, and of BCL7C in p53 inactivation and ovarian tumor suppression, open new avenues for precision medicine strategies, including epigenetic therapies and immunomodulatory approaches.

## Perspectives

- **Highlight the importance of the field:** In eukaryotic cells, DNA is packed into chromatin, and changes in chromatin organization are required for essential processes such as transcription, DNA repair, replication, and cell differentiation. These changes are partly controlled by the mSWI/SNF chromatin remodeling complexes, which uses ATP to reposition or remove nucleosomes and regulate DNA accessibility. Because mutations in mSWI/SNF subunits are frequently found in cancer and developmental disorders, understanding how these complexes function is an important challenge in chromatin biology. Among the recently characterized core subunits, BCL7 proteins have emerged as important components of mSWI/SNF complexes. Initially discovered through chromosomal translocations in lymphomas, BCL7 proteins are now recognized as structural components that directly interact with nucleosomes and contribute to stabilization of the ARP module. Understanding how BCL7 proteins contribute to chromatin remodeling is therefore important for understanding genome regulation in both normal and disease contexts.
- **Summary of the current thinking:** Recent biochemical and structural studies have greatly improved our understanding of BCL7 proteins. Cryo-EM studies showed that the conserved N-terminal region of BCL7 proteins binds the nucleosomal acidic patch through several conserved arginine residues, placing BCL7 among the few SWI/SNF subunits that directly contact histones. In addition, a conserved β-hairpin motif was shown to stabilize the ARP module through interactions with ACTL6A and β-actin, supporting an important structural role for BCL7 proteins within SWI/SNF complexes. Similar β-hairpin-based interaction modules are also found in plant and fungal chromatin remodeling complexes, suggesting that this structural mechanism has been conserved during evolution despite weak sequence similarity. Current evidence further suggests that the disordered C-terminal regions of BCL7 proteins may contribute to paralogue-specific regulatory functions through transient protein interactions and post-translational modifications. Their sequence composition may also support additional regulatory functions, although whether these include phase-separation-related mechanisms remains to be experimentally determined. Importantly, alterations in BCL7 genes are increasingly associated with hematological malignancies, solid tumors, and developmental disorders.
- **Comment on future directions:** Despite recent advances, many aspects of BCL7 biology remain poorly understood. An important objective will be to determine how BCL7 proteins are positioned within complete BAF and PBAF assemblies and how they cooperate or compete with other acidic-patch-binding subunits such as BAF47 during nucleosome remodeling. Future studies should also clarify how the conformational flexibility of the BCL7 N-terminal region contributes to nucleosome engagement in different SWI/SNF complexes, and whether the intrinsically disordered C-terminal regions regulate BCL7 activity through transient protein interactions or post-translational modifications. The structural similarity between BCL7 and ARP-associated factors such as Rtt102, DMAP1, and Eaf2 further suggests that conserved β-hairpin-mediated mechanisms may stabilize chromatin remodeling complexes across species. From a translational perspective, a better understanding of BCL7 molecular functions may also reveal clinically relevant vulnerabilities. The emerging links between BCL7A alterations and EZH2-associated vulnerabilities, BCL7B and immune regulation, and BCL7C and mutant p53 signaling suggest potential opportunities for biomarker development, patient stratification, and future targeted therapeutic strategies.

## Open Access

Open access for this article is provided via our Subscribe to Open programme, made possible through the support of our subscribing institutions. Learn more here: https://portlandpress.com/pages/open_access_options_and_prices#conditional.

## Abbreviations

ARP: actin-related protein
BCL7A: B-cell lymphoma 7 protein family member A
BDH: BCL-domain homologs
cBAF: BRG1-associated factor
cryo-EM: cryo-electron microscopy
DLBCL: diffuse large B-cell lymphoma
DNMAP1: DNA methyltransferase 1-associated protein 1
IGH: immunoglobulin heavy chain
ISWI: Imitation SWItch
mSWI/SNF: mammalian SWI
MYC: MYC proto-oncogene transcription factor
ncBAF: non-canonical BAF
NCP: Nucleosome core particle
NuA4: Nucleosome acetyltransferase of H4
PBAF: polybromo-associated BAF
SAGA: Spt–Ada–Gcn5 acetyltransferase
RSC: Remodels the Structure of Chromatin
SRCAP: SNF2-related CREBBP activator protein
TIP60: Tat-interactive protein of 60 kDa

## References

[1] Boehm T. and Rabbitts T.H. (1989) A chromosomal basis of lymphoid malignancy in man. Eur. J. Biochem. 185, 1–17 10.1111/j.1432-1033.1989.tb15074.x 2680485

[2] Jadayel D.M., Osborne L.R., Coignet L.J.A., Zani V.J., Tsui L.C., Scherer S.W. et al. (1998) The *BCL7* gene family: deletion of *BCL7B* Williams syndrome. Gene 224, 35–44 10.1016/S0378-1119(98)00514-9 9931421

[3] Zani V.J., Asou N., Jadayel D., Heward J.M., Shipley J., Nacheva E. et al. (1996) Molecular cloning of complex chromosomal translocation t(8;14;12)(q24.1;q32.3;q24.1) in a burkitt lymphoma cell line defines a new gene *(BCL7A)* with homology to caldesmon. Blood 87, 3124–3134 10.1182/blood.V87.8.3124.bloodjournal8783124 8605326

[4] Shain A.H. and Pollack J.R. (2013) The spectrum of SWI/SNF mutations, ubiquitous in human cancers. PloS ONE 8, e55119 10.1371/journal.pone.0055119 23355908 PMC3552954

[5] Kadoch C., Hargreaves D.C., Hodges C., Elias L., Ho L., Ranish J. et al. (2013) Proteomic and bioinformatic analysis of mammalian SWI/SNF complexes identifies extensive roles in human malignancy. Nat. Genet. 45, 592–601 10.1038/ng.2628 23644491 PMC3667980

[6] Middeljans E., Wan X., Jansen P.W., Sharma V., Stunnenberg H.G. and Logie C. (2012) SS18 together with animal-specific factors defines human BAF-type SWI/SNF complexes. PloS ONE 7, e33834 10.1371/journal.pone.0033834 22442726 PMC3307773

[7] Narlikar G.J., Sundaramoorthy R. and Owen-Hughes T. (2013) Mechanisms and functions of ATP-dependent chromatin-remodeling enzymes. Cell 154, 490–503 10.1016/j.cell.2013.07.011 23911317 PMC3781322

[8] Candela-Ferre J., Pérez-Alemany J., Diego-Martin B., Pandey V., Wohlschlegel J., Lozano-Juste J. et al. (2025) Plant BCL-DOMAIN HOMOLOG proteins play a conserved role in SWI/SNF complex stability. Proc. Natl. Acad. Sci. U.S.A. 122, e2413346122 10.1073/pnas.2413346122 39823297 PMC11761322

[9] Stachula P., Kapela K., Malecka E., Jaronczyk K., Patryn J., Siwirykow N. et al. (2023) BRM complex in arabidopsis adopts ncBAF-like composition and requires BRD subunits for assembly and stability. Int. J. Mol. Sci. 24, 3917 10.3390/ijms24043917 36835328 PMC9967331

[10] Jones D.T. and Cozzetto D. (2015) DISOPRED3: precise disordered region predictions with annotated protein-binding activity. Bioinformatics 31, 857–863 10.1093/bioinformatics/btu744 25391399 PMC4380029

[11] Abramson J., Adler J., Dunger J., Evans R., Green T., Pritzel A. et al. (2024) Accurate structure prediction of biomolecular interactions with AlphaFold 3. Nature 630, 493–500 10.1038/s41586-024-07487-w 38718835 PMC11168924

[12] Martin F., Kazrani A.A., Lafouge J., Diaz-Jimenez D.M., Siebert S., Fabbro-Burtschell L. et al. (2025) Structure of the nucleosome-bound human BCL7A. Nucleic. Acids. Res. 53, gkaf273 10.1093/nar/gkaf273 40207634 PMC11983133

[13] Jumper J., Evans R., Pritzel A., Green T., Figurnov M., Ronneberger O. et al. (2021) Highly accurate protein structure prediction with AlphaFold. Nature 596, 583–589 10.1038/s41586-021-03819-2 34265844 PMC8371605

[14] Varadi M., Anyango S., Deshpande M., Nair S., Natassia C., Yordanova G. et al. (2022) AlphaFold protein structure database: massively expanding the structural coverage of protein-sequence space with high-accuracy models. Nucleic. Acids. Res. 50, D439–D444 10.1093/nar/gkab1061 34791371 PMC8728224

[15] Varadi M., Bertoni D., Magana P., Paramval U., Pidruchna I., Radhakrishnan M. et al. (2024) AlphaFold protein structure database in 2024: providing structure coverage for over 214 million protein sequences. Nucleic Acids Res. 52, D368–D375 10.1093/nar/gkad1011 37933859 PMC10767828

[16] Xue J., Tian K., Xu X., Feng Y., Yu M., Hao M. et al. (2026) BCL7A’s arginine anchor links nucleosome recognition to chromatin remodeling and diffuse large B-cell lymphoma tumor suppression. Protein Cell 17, 452–470 10.1093/procel/pwaf114 41485079 PMC13161475

[17] Mashtalir N., D’Avino A.R., Michel B.C., Luo J., Pan J., Otto J.E. et al. (2018) Modular organization and assembly of SWI/SNF family chromatin remodeling complexes. Cell 175, 1272.e20–1288.e20 10.1016/j.cell.2018.09.032 30343899 PMC6791824

[18] He S., Wu Z., Tian Y., Yu Z., Yu J., Wang X. et al. (2020) Structure of nucleosome-bound human BAF complex. Science 367, 875–881 10.1126/science.aaz9761 32001526

[19] Wang L., Yu J., Yu Z., Wang Q., Li W., Ren Y. et al. (2022) Structure of nucleosome-bound human PBAF complex. Nat Commun. 13, 7644 10.1038/s41467-022-34859-5 36496390 PMC9741621

[20] Yuan J., Chen K., Zhang W. and Chen Z. (2022) Structure of human chromatin-remodelling PBAF complex bound to a nucleosome. Nature 605, 166–171 10.1038/s41586-022-04658-5 35477757

[21] Mashtalir N., Suzuki H., Farrell D.P., Sankar A., Luo J., Filipovski M. et al. (2020) A structural model of the endogenous human BAF complex informs disease mechanisms. Cell 183, 802.e24–817.e24 10.1016/j.cell.2020.09.051 33053319 PMC7717177

[22] Chen K., Du L., Liu Y., Chen M. and Chen Z. (2025) ncBAF recognizes the nucleosome through BCL7A in chromatin remodeling. Cell Discov. 11, 102 10.1038/s41421-025-00858-1 41402274 PMC12708618

[23] Sun F., Zou B., Li H., Xu C., Luo Q., Wang C. et al. (2026) Structural basis for BCL7B-mediated ncBAF-nucleosome engagement. Nucleic. Acids. Res. 54, gkag092 10.1093/nar/gkag092 41657245 PMC12884074

[24] McGinty R.K. and Tan S. (2015) Nucleosome structure and function. Chem. Rev. 115, 2255–2273 10.1021/cr500373h 25495456 PMC4378457

[25] McGinty R.K. and Tan S. (2021) Principles of nucleosome recognition by chromatin factors and enzymes. Curr. Opin. Struct. Biol. 71, 16–26 10.1016/j.sbi.2021.05.006 34198054 PMC8648869

[26] Qu K., Chen K., Wang H., Li X. and Chen Z. (2022) Structure of the NuA4 acetyltransferase complex bound to the nucleosome. Nature 610, 569–574 10.1038/s41586-022-05303-x 36198799

[27] Baker R.W., Reimer J.M., Carman P.J., Turegun B., Arakawa T., Dominguez R. et al. (2021) Structural insights into assembly and function of the RSC chromatin remodeling complex. Nat. Struct. Mol. Biol. 28, 71–80 10.1038/s41594-020-00528-8 33288924 PMC7855068

[28] Dao H.T., Dul B.E., Dann G.P., Liszczak G.P. and Muir T.W. (2020) A basic motif anchoring ISWI to nucleosome acidic patch regulates nucleosome spacing. Nat. Chem. Biol. 16, 134–142 10.1038/s41589-019-0413-4 31819269 PMC6982587

[29] Morgan M.T., Haj-Yahya M., Ringel A.E., Bandi P., Brik A. and Wolberger C. (2016) Structural basis for histone H2B deubiquitination by the SAGA DUB module. Science 351, 725–728 10.1126/science.aac5681 26912860 PMC4863942

[30] Turegun B., Kast D.J. and Dominguez R. (2013) Subunit Rtt102 controls the conformation of the Arp7/9 heterodimer and its interactions with nucleotide and the catalytic subunit of SWI/SNF remodelers. J. Biol. Chem. 288, 35758–35768 10.1074/jbc.M113.514083 24189066 PMC3861627

[31] Musselman C.A. and Kutateladze T.G. (2021) Characterization of functional disordered regions within chromatin-associated proteins. iScience 24, 102070 10.1016/j.isci.2021.102070 33604523 PMC7873657

[32] Theillet F.X., Smet-Nocca C., Liokatis S., Thongwichian R., Kosten J., Yoon M.K. et al. (2012) Cell signaling, post-translational protein modifications and NMR spectroscopy. J. Biomol. NMR 54, 217–236 10.1007/s10858-012-9674-x 23011410 PMC4939263

[33] Rippe K. (2022) Liquid–liquid phase separation in chromatin. Cold Spring Harb. Perspect. Biol. 14, a040683 10.1101/cshperspect.a040683 34127447 PMC8805649

[34] Jiao L. and Liu X. (2015) Structural basis of histone H3K27 trimethylation by an active polycomb repressive complex 2. Science 350, aac4383 10.1126/science.aac4383 26472914 PMC5220110

[35] Dietrich N., Trotter K., Ward J.M. and Archer T.K. (2023) BRG1 HSA domain interactions with BCL7 proteins are critical for remodeling and gene expression. Life Sci. Alliance 6, e202201770 10.26508/lsa.202201770 36801810 PMC9939006

[36] Chakraborty C., Talluri S., Binder M., Morelli E., Mayoral J.E., Derebail S. et al. (2025) Loss of BCL7A permits IRF4 transcriptional activity and cellular growth in multiple myeloma. Blood 146, 104–114 10.1182/blood.2024026588 40090008 PMC12782957

[37] Wischhof L., Lee H., Tutas J., Overkott C., Tedt E., Stork M. et al. (2022) BCL7A‐containing SWI/SNF/BAF complexes modulate mitochondrial bioenergetics during neural progenitor differentiation. EMBO J. 41, e110595 10.15252/embj.2022110595 36305367 PMC9713712

[38] Haussmann I.U., White K. and Soller M. (2008) Erect wing regulates synaptic growth in *Drosophila* by integration of multiple signaling pathways. Genome Biol. 9, R73 10.1186/gb-2008-9-4-r73 18419806 PMC2643944

[39] Uehara T., Kage-Nakadai E., Yoshina S., Imae R. and Mitani S. (2015) The tumor suppressor BCL7B functions in the Wnt signaling pathway. PLos Genet. 11, e1004921 10.1371/journal.pgen.1004921 25569233 PMC4287490

[40] Baliñas-Gavira C., Rodríguez M.I., Andrades A., Cuadros M., Álvarez-Pérez J.C., Álvarez-Prado Á.F. et al. (2020) Frequent mutations in the amino-terminal domain of BCL7A impair its tumor suppressor role in DLBCL. Leukemia 34, 2722–2735 10.1038/s41375-020-0919-5 32576963

[41] Forbes S.A., Beare D., Gunasekaran P., Leung K., Bindal N., Boutselakis H. et al. (2015) COSMIC: exploring the world’s knowledge of somatic mutations in human cancer. Nucleic. Acids. Res. 43, D805–D811 10.1093/nar/gku1075 25355519 PMC4383913

[42] Chakraborty C., Talluri S., Morelli E., Derebail S., Yao Y., Xu Y. et al. (2021) Universally observed loss of BCL7A allows activation of IRF4 and its transcriptional activity in multiple myeloma cells. Blood 138, 2667 10.1182/blood-2021-153462

[43] Reddy A., Zhang J., Davis N.S., Moffitt A.B., Love C.L., Waldrop A. et al. (2017) Genetic and functional drivers of diffuse large B cell lymphoma. Cell 171, 481.e15–494.e15 10.1016/j.cell.2017.09.027 28985567 PMC5659841

[44] Sun Z., Sun L., He M., Pang Y., Yang Z. and Wang J. (2019) Low BCL7A expression predicts poor prognosis in ovarian cancer. J. Ovarian Res. 12, 41 10.1186/s13048-019-0518-0 31077237 PMC6511192

[45] Maura F., Ziccheddu B., Xiang J.Z., Bhinder B., Rosiene J., Abascal F. et al. (2023) Molecular evolution of classic hodgkin lymphoma revealed through whole-genome sequencing of hodgkin and reed sternberg cells. Blood Cancer Discov. 4, 208–227 10.1158/2643-3230.BCD-22-0128 36723991 PMC10150291

[46] Patiño-Mercau J.R., Baliñas-Gavira C., Andrades A., Benitez-Cantos M.S., Rot A.E., Rodriguez M.I. et al. (2023) BCL7A is silenced by hypermethylation to promote acute myeloid leukemia. Biomark Res. 11, 32 10.1186/s40364-023-00472-x 36941700 PMC10026484

[47] Higuchi S., Suehiro Y., Izuhara L., Yoshina S., Hirasawa A. and Mitani S. (2023) BCL7B, a SWI/SNF complex subunit, orchestrates cancer immunity and stemness. BMC Cancer 23, 811 10.1186/s12885-023-11321-3 37648998 PMC10466690

[48] Liu J., Gao L., Ji B., Geng R., Chen J., Tao X. et al. (2021) BCL7A as a novel prognostic biomarker for glioma patients. J. Transl. Med. 19, 335 10.1186/s12967-021-03003-0 34362400 PMC8348860

[49] Taniuchi K., Furihata M., Naganuma S., Dabanaka K., Hanazaki K. and Saibara T. (2018) BCL7B, a predictor of poor prognosis of pancreatic cancers, promotes cell motility and invasion by influencing CREB signaling. Am. J. Cancer. Res. 8, 387–404 29636996 PMC5883091

[50] Taniuchi K., Furihata M., Naganuma S., Sakaguchi M. and Saibara T. (2019) Overexpression of PODXL/ITGB1 and BCL7B/ITGB1 accurately predicts unfavorable prognosis compared to the TNM staging system in postoperative pancreatic cancer patients. PloS ONE 14, e0217920 10.1371/journal.pone.0217920 31166991 PMC6550449

[51] Yang D., Li H., Chen Y., Li C., Ren W. and Huang Y. (2022) A pan-cancer analysis of the oncogenic role of BCL7B: A potential biomarker for prognosis and immunotherapy. Front Genet. 13, 906174 10.3389/fgene.2022.906174 35910232 PMC9334570

[52] Huang C., Hao Q., Shi G., Zhou X. and Zhang Y. (2021) BCL7C suppresses ovarian cancer growth by inactivating mutant p53. J. Mol. Cell Biol. 13, 141–150 10.1093/jmcb/mjaa065 33306126 PMC8104935

[53] Konstantinidou M. and Arkin M.R. (2024) Molecular glues for protein–protein interactions: Progressing toward a new dream. Cell Chem. Biol. 31, 1064–1088 10.1016/j.chembiol.2024.04.002 38701786 PMC11193649

[54] Wu K., Jiang H., Hicks D.R., Liu C., Muratspahić E., Ramelot T.A. et al. (2025) Design of intrinsically disordered region binding proteins. Science 389, eadr8063 10.1126/science.adr8063 40674483 PMC12949689

[55] Watson J.L., Juergens D., Bennett N.R., Trippe B.L., Yim J., Eisenach H.E. et al. (2023) *De novo* design of protein structure and function with RFdiffusion. Nature 620, 1089–1100 10.1038/s41586-023-06415-8 37433327 PMC10468394

[56] Li C., Smirnova E., Schnitzler C., Crucifix C., Concordet J.P., Brion A. et al. (2024) Structure of the human TIP60-C histone exchange and acetyltransferase complex. Nature 635, 764–769 10.1038/s41586-024-08011-w 39260417 PMC11578891

[57] Yu J., Sui F., Gu F., Li W., Yu Z., Wang Q. et al. (2024) Structural insights into histone exchange by human SRCAP complex. Cell Discov. 10, 15 10.1038/s41421-023-00640-1 38331872 PMC10853557

